# Inhibition of neuronal necroptosis mediated by RIP1/RIP3/MLKL provides neuroprotective effects on kaolin‐induced hydrocephalus in mice

**DOI:** 10.1111/cpr.13108

**Published:** 2021-08-09

**Authors:** Chang Liu, Yaxing Chen, Wenyao Cui, Yi Cao, Long Zhao, Haoxiang Wang, Xiaoyin Liu, Shuangmin Fan, Keru Huang, Aiping Tong, Liangxue Zhou

**Affiliations:** ^1^ Department of Neurosurgery West China Medical School West China Hospital Sichuan University Chengdu China; ^2^ Department of Neurosurgery Chengdu Second People's hospital Chengdu China; ^3^ State Key Laboratory of Biotherapy West China Medical School Sichuan University Chengdu China

**Keywords:** cortex, hippocampus, hydrocephalus, necroptosis, neuroinflammation

## Abstract

**Objectives:**

Necroptosis is widespread in neurodegenerative diseases. Here, we examined necroptosis in the hippocampus and cortex after hydrocephalus and found that a necroptosis pathway inhibitor alleviates necroptosis and provides neuroprotective effects.

**Materials and methods:**

Hydrocephalus was induced in C57BL/6 mice by kaolin. Haematoxylin and eosin (HE), Nissl, PI and Fluoro‐Jade B (FJB) staining were used for general observations. Phosphorylated receptor‐interacting protein kinase 3 (p‐RIP3) and phosphorylated mixed lineage kinase domain‐like (p‐MLKL) were measured by Western blotting and immunohistochemistry. Scanning electron microscopy (SEM) was used to observe ependymal cilia. Magnetic resonance imaging (MRI) and the Morris water maze (MWM) test were used to assess neurobehavioral changes. Immunofluorescence was used to detect microglial and astrocyte activation. Inflammatory cytokines were measured by Western blotting and RT‐PCR.

**Results:**

Obvious pathological changes appeared in the hippocampus and cortex after hydrocephalus, and expression of the necroptosis markers p‐RIP3, p‐MLKL and inflammatory cytokines increased. Necrostatin‐1 (Nec‐1) and GSK872 reduced necrotic cell death, attenuated p‐RIP3 and p‐MLKL levels, slightly improved neurobehaviours and inhibited microglial and astrocyte activation and inflammation.

**Conclusions:**

RIP1/RIP3/MLKL mediates necroptosis in the cortex and hippocampus in a hydrocephalus mouse model, and Nec‐1 and GSK872 have some neuroprotective effects.

## Graphical abstract

After hydrocephalus, RIP1 recruits and promotes RIP3 phosphorylation. Then, a body consisting of RIP1, RIP3 and MLKL is formed. MLKL is phosphorylated by p‐RIP3, located at position 357 of threonine and position 358 of serine, and is considered to be the executor of necroptosis. p‐MLKL finally leads to the exposure of cells by changing permeabilization in plasma membrane and result in necrosis of the cell. However, the effects were improved after the use necroptosis inhibitors Nec‐1 and GSK872.

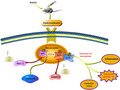

## 1 INTRODUCTION

Hydrocephalus is a disease in which excessive secretion, circulation and absorption of cerebrospinal fluid (CSF) are impaired by craniocerebral diseases, resulting in increased intracranial CSF levels, expansion of the ventricular system and progressive expansion of the subarachnoid space.^1^ Hydrocephalus can be primary or secondary to trauma, spontaneous bleeding, infection and tumours and can also be comorbid with neurological diseases such as dementia, Alzheimer's disease and Parkinson's disease.^2^, ^3^, ^4^ Hydrocephalus is a great challenge in neurosurgery because of its complicated aetiology, unclear mechanism, difficult treatment and many complications. Therefore, it is important to find a suitable and effective treatment for hydrocephalus.

Apoptosis and necrosis are two major types of cell death and are traditional classifications of cell death modalities. In recent years, this viewpoint has been challenged by the discovery of other types of programmed cell death, such as necroptosis, ferroptosis and pyroptosis.^5^, ^6^, ^7^ In vivo evidence supports that necroptosis may have important functions in many human inflammatory diseases.^8^ Necroptosis can be defined as follows: upon stimulation by death signals, cells upregulate receptor‐interacting protein kinase 1 (RIP1) and receptor‐interacting protein kinase 3 (RIP3), which activate a signalling cascade involving mixed lineage kinase domain‐like protein (MLKL) phosphorylation, which contributes to a conformational change in the pseudokinase domain and ultimately changes the plasm membrane permeability, resulting in necrosis.^9^, ^10^, ^11^ RIP1 activation has been shown to mediate multiple alternative cell death mechanisms, including RIPK1‐dependent apoptosis (RDA) under apoptosis‐proficient conditions and necroptosis under apoptosis‐deficient conditions.^12^, ^13^ Necrostatin‐1 (Nec‐1) is a highly specific RIP1 kinase inhibitor, and numerous studies have demonstrated its neuroprotective effects.^14^ Moreover, necroptosis has been shown to be dependent on RIP3 and MLKL.^15^ The selective RIP3 inhibitor GSK872 can block necroptosis in human and murine cells, and in general, both Nec‐1 and GSK872 inhibit necroptosis and inflammation.^16^, ^17^ Necroptosis has been reported in various models of neurological diseases, such as amyotrophic lateral sclerosis, ischaemic brain injury, intracerebral haemorrhage and traumatic brain injury (TBI).^18^, ^19^, ^20^, ^21^ However, whether necroptosis occurs in hydrocephalus remains unknown.

In the present study, we demonstrated that RIP1/RIP3/MLKL‐mediated necroptosis contributes to necrotic cell death after kaolin‐induced hydrocephalus in mice. We further showed that the RIP1 kinase inhibitor Nec‐1 and the RIP3 kinase inhibitor GSK872 exert neuroprotective effects against necroptosis by inhibiting the RIP1/RIP3/MLKL pathway and inflammation after hydrocephalus.

## 2 MATERIALS AND METHODS

### 2.1 Experiment animals

One hundred and forty C57BL/6 mice (weighing 18‐23 g) were provided by the Laboratory Animal Center of Sichuan University for use in this study. The mice were housed under controlled, pathogen‐free conditions with free access to pellet food and water. All procedures were strictly performed according to the animal use protocols, which were approved by the Animal Care and Use Committee of Sichuan University.

### 2.2 Kaolin‐induced hydrocephalus models and drug administration

The hydrocephalus model was induced via kaolin injection into the cisterna magna.^22^ The mice were anaesthetized with pentobarbital (40 mg/kg intraperitoneally). The skin was incised along the midline of the neck, the muscles were separated, and the cisterna magna was exposed under aseptic conditions. Then, a 30‐gauge needle (Hamilton, Switzerland), which was custom bent to 30°‐45°, was inserted into the cisterna magna. A 6‐μl volume of a sterile kaolin suspension (100 mg/mL, Sigma‐Aldrich) in 0.9% sterile saline was injected for approximately 30 seconds. The experimental groups were divided into the sham group, hydrocephalus group, hydrocephalus+Nec‐1 group (Nec‐1 group) and hydrocephalus+GSK872 group (GSK872 group). The specific necroptosis inhibitors Nec‐1 (Selleck) and GSK872 (Merck Millipore, Billerica, MA, USA) were dissolved in DMSO and diluted to a final concentration of 25 mM.^23^ A total of 1 μL of Nec‐1 and 1 μL of GSK872 were injected four times into the lateral ventricles (coordinates relative to the bregma: 0.5 mm posterior, 1 mm lateral, 2.2 mm deep) of the mice once per week after the kaolin injection.

### 2.3 Cell death assays

The mice were deeply anaesthetized and perfused with 4% paraformaldehyde in 0.1 M phosphate‐buffered saline (PBS), and the brains were removed. For HE staining, the brain tissues were fixed in paraformaldehyde, embedded in paraffin and cut into sections. The tissue sections were dewaxed, hydrated and stained with haematoxylin for 10 minutes. After being rinsed with tap water, 0.5% eosin staining solution was added dropwise, and the sections were stained at room temperature for 1 hour. The slides were rinsed with tap water for several seconds, dehydrated, sealed and observed under a light microscope. For Nissl staining, paraffin‐embedded sections were dewaxed and rehydrated. Then, the slides were placed in 1:1 alcohol:chloroform overnight and rinsed with distilled water, and the stained slices were placed in 1% cresol purple solution (heated in an oven at 37‐50℃) for 5‐10 minutes and rinsed quickly in distilled water. The slides were differentiated in 95% ethanol for 20‐30 minutes and examined under a microscope. Neuron apoptosis in the cortex and hippocampus was analysed using TUNEL staining according to the manufacturer's protocol (Servicebio). Some sections were then counterstained with 4’,6‐diamidino‐2‐phenyl‐indole (DAPI, Thermo Fisher Scientific). For PI staining, at 28 days after kaolin injection, PI (Sigma‐Aldrich) was diluted to 10 mg/mL in sterile saline, and 1 µL was injected intracerebroventricularly into each mouse 1 hour before euthanasia. PI staining was observed in frozen brain sections, which were covered with DAPI for 5 minutes and photographed immediately. For Fluoro‐Jade B (FJB) staining, the brain slices were soaked in 1% sodium hydroxide with 80% alcohol, followed by soaking in 70% alcohol. The slides were incubated with 0.06% potassium permanganate at room temperature for 10 minutes and washed with distilled water. Then, the slides were incubated in FJB solution (Merck Millipore) at room temperature for 20 minutes and observed under a microscope.

### 2.4 Western blotting, immunohistochemistry, immunofluorescence analysis

Total proteins were harvested from the cortex and hippocampus. The proteins were transferred to polyvinylidene fluoride membranes (Millipore) that were blocked with 5% bovine serum albumin (BSA) for 1 hour at room temperature and incubated overnight with the following primary antibodies at 4℃: anti‐p‐RIP3 (Abcam), anti‐p‐MLKL (Abcam), anti‐tumour necrosis factor‐α (TNF‐α) (Abcam), anti‐IL‐1β (Abcam), anti‐IL‐6 (Abcam) and anti‐β‐actin (Cell Signaling Technology). After incubation with secondary antibodies (Solarbio), the positive bands were visualized using an ECL substrate (Solarbio). For immunohistochemical analysis, brain tissue sections were incubated overnight with the following primary antibodies at 4℃: anti‐p‐RIP3 (Abcam) and anti‐p‐MLKL (Abcam). Subsequently, a PV9000 kit (ZSGB‐BIO) was used for follow‐up. For immunofluorescence analysis, brain tissues were blocked with goat serum for 1 hour and incubated with primary antibodies against p‐RIP3 (Abcam), p‐MLKL (Abcam), NeuN (Abcam), Iba‐1 (Wako; Servicebio) and GFAP (Servicebio) overnight at 4℃. Then, the tissues were incubated with Alexa Fluor® 594 goat anti‐rabbit IgG and Alexa Fluor® 488 goat anti‐mouse IgG secondary antibodies (Thermo Fisher Scientific). Nuclei were counterstained with DAPI (Thermo Fisher Scientific).

### 2.5 Magnetic resonance imaging (MRI) and the Evans ratio

Mice were anaesthetized using a 2% isoflurane‐air mixture during MRI. MRI was performed in a 7.0‐Tesla MR scanner (Bruker BioSpec 70/30 MRI) at 28 days after kaolin injection. The following parameters were chosen: field of view, 35 × 35 mm; matrix, 256 × 256; slice thickness, 1 mm; echo time, 2.5 ms; and repetition time, 100 ms The Evans ratio is the ratio of the greatest width of the lateral ventricles to the greatest width of the brain. An Evans ratio>0.3 can be used to diagnose hydrocephalus.

### 2.6 Morris water maze test

Learning and spatial memory were assessed by the Morris water maze (MWM) test 23‐28 days after kaolin injection, and mice in the sham group were evaluated at the same time. The mice were placed in a pool of water (25℃) with a depth of 50 cm and a diameter of 200 cm, which was made opaque with titanium dioxide. The pool was divided into four equally sized quadrants, and a submerged platform (1 ~ 1.5 cm; 6 cm × 6 cm below the water surface) was fixed in the target quadrant. The mice were allowed to find the submerged platform within 180 seconds. Then, a random starting position was used for 5 days of training in the four quadrants. The latency times of 4 trials were recorded and averaged each day. On the sixth day of testing, after the submerged platform was removed, the mice completed a 120‐second probe trial. Platform crossing times, distances travelled and times in the target quadrant were calculated.

### 2.7 Scanning electron microscopy (SEM)

After soaking in 2.5% glutaraldehyde for 12 hour, the target sections were removed from the brain tissue. The selected brain tissue blocks were placed into 0.1 mol/L phosphate buffer overnight, followed by gradient alcohol dehydration and isoamyl acetate replacement. The specimens were then critical‐point dried in CO2, mounted on SEM stubs and sputter‐coated with gold‐palladium. Then, the surfaces of the brain slices were examined with an AMR‐1400 scanning electron microscope (Phillips) at an accelerating voltage of 20 kV.

### 2.8 Real‐time quantitative polymerase chain reaction (RT‐PCR)

Total RNA was extracted from the cortex and hippocampus using TRIzol reagent (Invitrogen, United States). Complementary DNA synthesis was performed using a reverse transcriptase kit (Invitrogen). Real‐time PCR was performed using a PowerUp^TM^ SYBRTM Green Master Mix kit (Thermo Fisher). RNA sequences are shown in Table 1.

**TABLE 1:** Primers designed for qRT‐PCR validation

Gene	Primer
IL‐1β	**F** CTACAGGCTCCGAGATGAACAA **R** TTCTTCTTTGGGTATTGCTTGG
IL‐6	**F** AGTTGTGCAATGGCAATTCTGA **R** CTCTGAAGGACTCTGGCTTTGTC
TNF‐α	**F** CCCTCACACTCACAAACCACC **R** CTTTGAGATCCATGCCGTTG
GAPDH	**F** ATGGCAAGTTCAAAGGCACAGTCA **R** TGGGGGCATCAGCAGAAGG

### 2.9 Statistical analysis

Statistical analysis was performed using GraphPad Prism software (GraphPad Software). All data are presented as the mean ± SEM. Statistical differences among groups were analysed using Student's *t*‐test, and a P‐value <0.05 was considered statistically significant.

## 3 RESULTS

### 3.1 Weight loss, destruction of ependymal cilia and pathological damage in hippocampal and cortex after kaolin‐induced hydrocephalus

**FIGURE 1:**
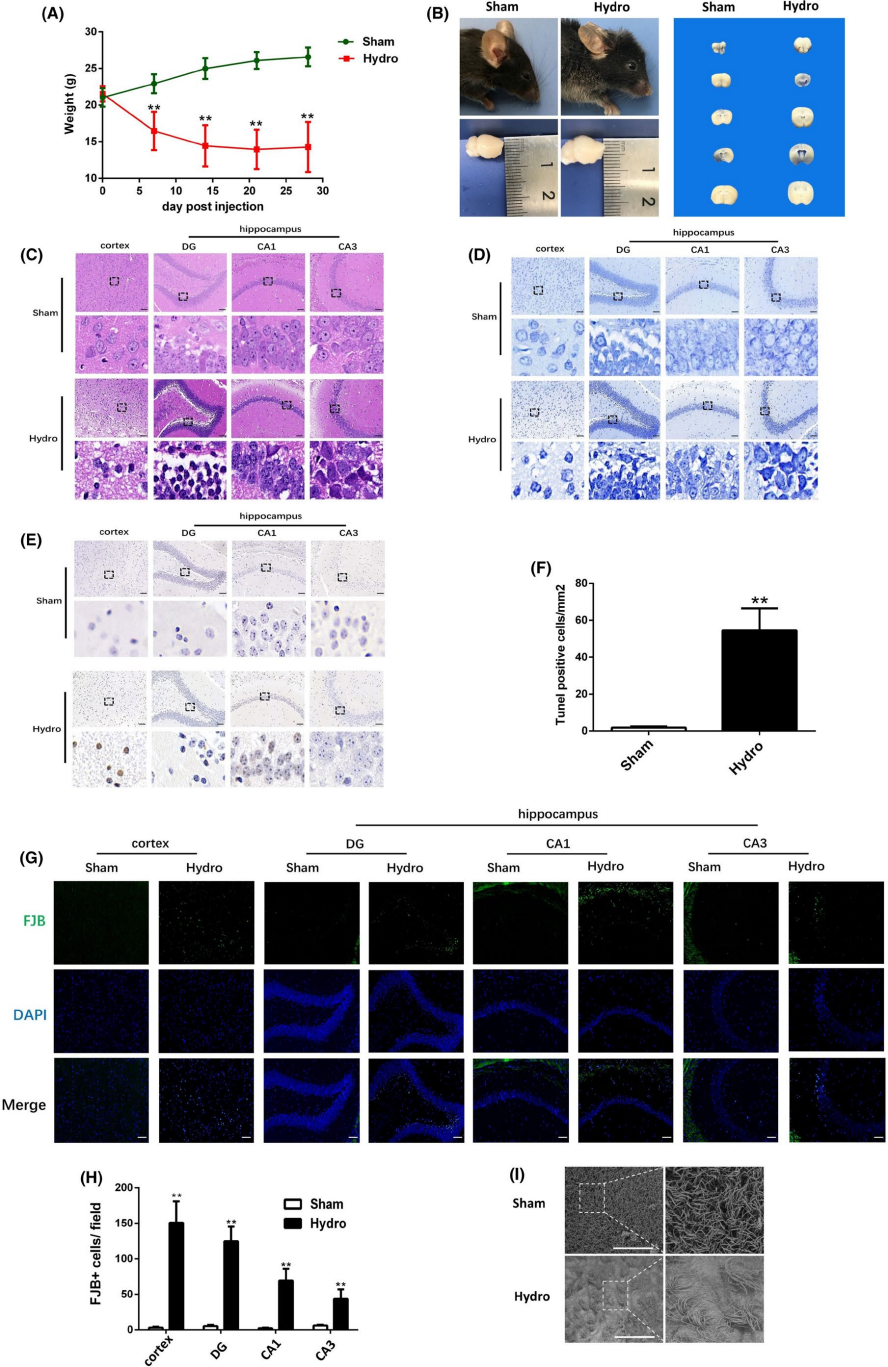
General observation of hippocampus and cortex in sham group and hydrocephalus group. (A) Changes of body weight in sham group and hydrocephalus group at 7, 14, 21, 28 days (B) and brain tissue sections to observe the changes of the size of lateral ventricle. Representative HE‐stained (C), Nissl‐stained (D) and TUNEL‐stained (E) images of the cortex and hippocampal DG, CA1 and CA3 regions in brain sections from sham group and hydrocephalus group. Bar = 50 μm. Below panel: enlargements of images in the boxes in the up panel. (F) Quantitative analysis shows that TUNEL‐positive cells in cortex in hydrocephalus group were increased compared with sham group. (G) Representative images of FJB (green) in sham group and hydrocephalus group. Nuclei were counterstained with DAPI (blue). Bar=50 μm. (H) Quantitative analysis shows that FJB‐positive cells in hydrocephalus group were increased compared with sham group. (I) Representative image of scanning electron microscopy to observe ependymal cilia in sham group and hydrocephalus group. Bar = 40 μm. n = 6 animals per group. Data are expressed as the mean ± SEM; statistical differences among groups were analysed using Student's *t*‐test; **P < .01 vs. sham

Body weight was significantly lower in the hydrocephalus group than the sham group at different time points (Figure 1A). We observed significant hydrocephalus in the mice 28 days after model establishment (Figure 1B). HE staining revealed cell body shrinkage and nuclear edge aggregation in the cortex and hippocampus in the hydrocephalus group (Figure 1C). The Nissl staining results confirmed that hydrocephalus reduced the number of surviving neurons compared with the sham group (Figure 1D). In addition, we found that there were few TUNEL‐positive cells in the hippocampus after hydrocephalus, whereas TUNEL‐positive cells were obvious in the cortex (Figure 1E,F). FJB staining further confirmed that hydrocephalus reduced the numbers of surviving neurons in the cortex and hippocampus compared with the sham group (Figure 1G,H). Moreover, SEM showed that ependymal cilia in the hydrocephalus group were significantly destroyed and sparsely distributed (Figure 1I).

### 3.2 Necroptosis, necrotic cell death and inflammatory cytokines are increased in the cortex and hippocampus after kaolin‐induced hydrocephalus

**FIGURE 2:**
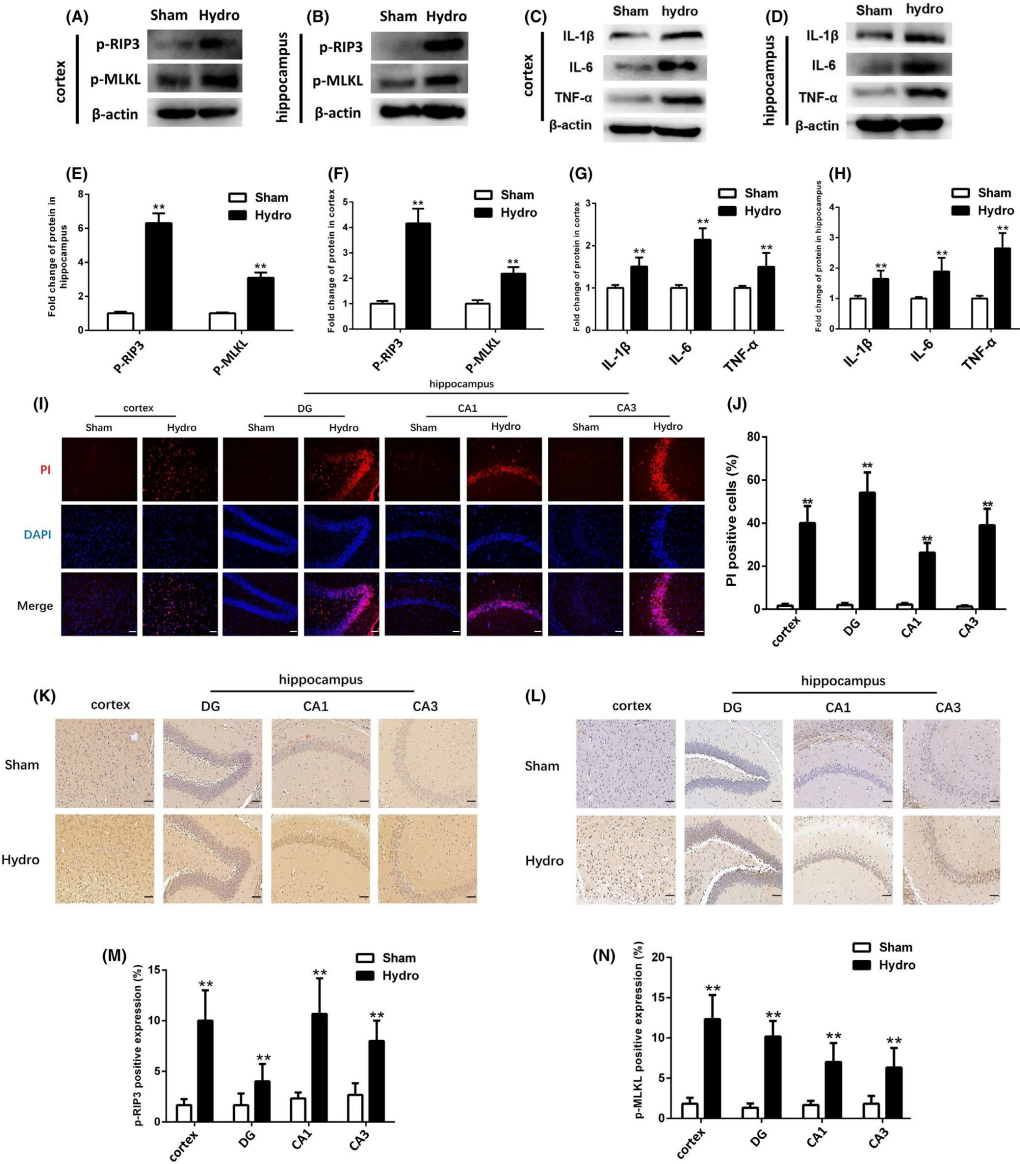
Observation the expression of necroptosis markers, inflammatory cytokines and necrotic cell death in cortex and hippocampal after hydrocephalus. (A‐D) Representative Western bands showing the protein expression of p‐RIP3, p‐MLKL, IL‐1β, IL‐6 and TNF‐α in cortex and hippocampus of sham group and hydrocephalus group at 28 days after kaolin injection. (E‐H) Quantitative analyses of p‐RIP3, p‐MLKL, IL‐1β, IL‐6 and TNF‐α protein levels in the cortex and hippocampus in sham group and hydrocephalus group. (I) Representative fluorescence micrographs of PI labelling (Red) in sham and hydrocephalus groups at 28 days after kaolin injection. Nuclei were counterstained with DAPI (blue). Bar = 50μm. (J) the number of PI‐positive cells increased in cortex and hippocampus of hydrocephalus group compared with sham group. (K, L) Representative images of immunohistochemistry staining of p‐RIP3 and p‐MLKL in cortex and hippocampal DG, CA1 and CA3 regions. Bar = 50μm. (M, N) Quantitative analyses of p‐RIP3 and p‐MLKL‐positive expression in cortex and hippocampus DG, CA1, CA3 region in sham group and hydrocephalus group. n = 6 animals per group. Data are expressed as the mean ± SEM; statistical differences among groups were analysed using Student's *t*‐test; **P < .01 vs. sham

Western blotting showed that the expression levels of p‐RIP3 and p‐MLKL in the hippocampus and cortex were increased at 28 days compared with the sham group (Figure 2A,B,E,F). Similarly, the immunohistochemical results showed that the expression of p‐RIP3 and p‐MLKL in the hippocampus and the cortex were higher in the hydrocephalus group than in the sham group (Figure 2K‐N). In addition, necrotic cell death was examined by PI labelling. The results showed that compared with those in the sham group, hydrocephalus triggered significant increases in the numbers of PI‐positive cells in the cortex and hippocampus (Figure 2I,J). Furthermore, we assessed several proinflammatory cytokines, such as IL‐1β, IL‐6 and TNF‐α, by Western blotting, and the results showed that the expression levels of IL‐1β, IL‐6 and TNF‐α in the cortex and hippocampus were increased in the hydrocephalus group compared with the sham group at 28 days (Figure 2C,D,G,H).

### 3.3 Phosphorylated RIP3 and MLKL are mainly expressed by neuronal cells in the cortex and hippocampus after kaolin‐induced hydrocephalus

**FIGURE 3:**
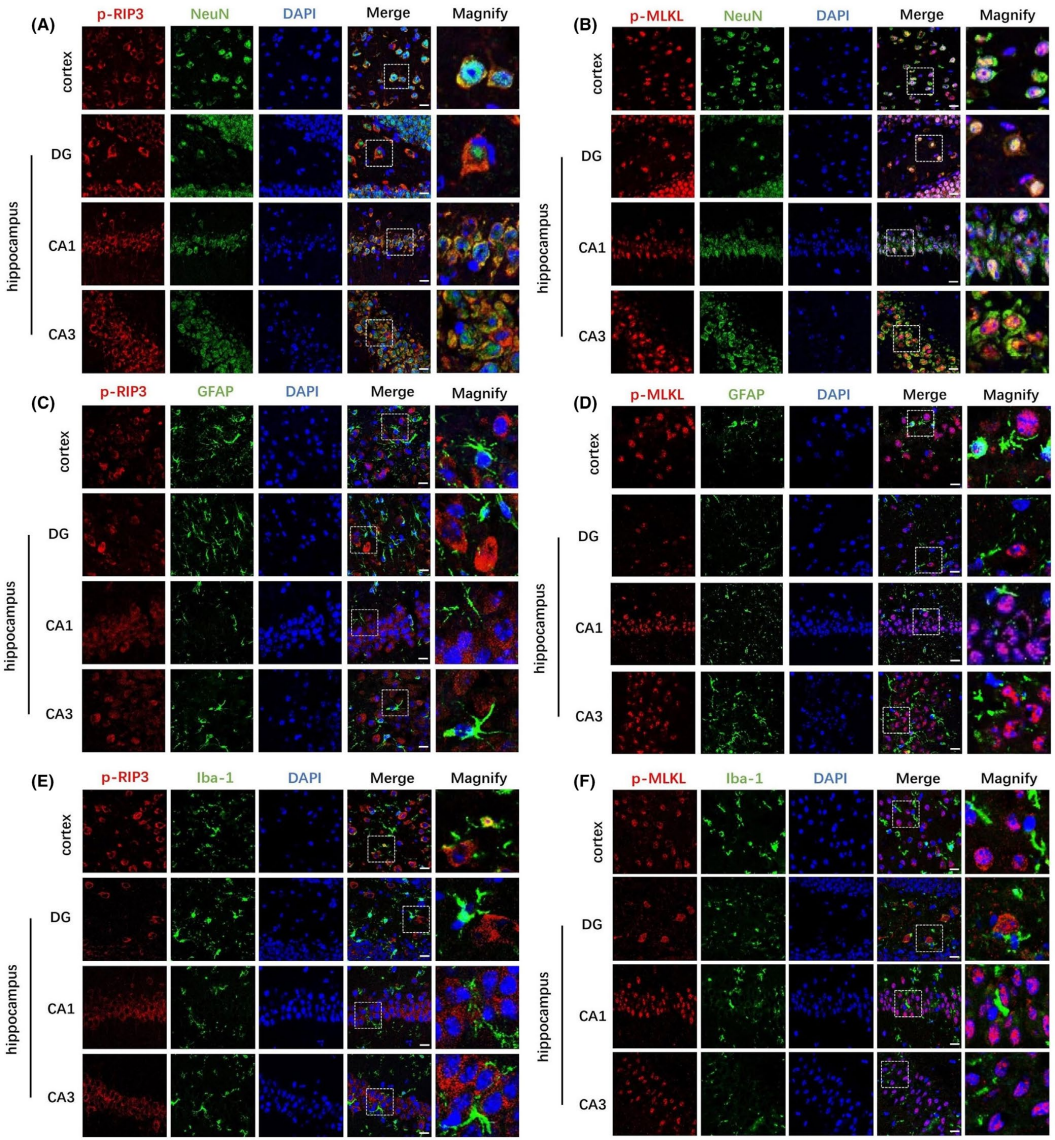
Double immunofluorescence staining of three types of cells with necroptosis markers p‐RIP3, p‐MLKL. (A, B) Representative immunofluorescence staining images of p‐RIP3, p‐MLKL (red) and NeuN (green) in cortex and hippocampal DG, CA1 and CA3 regions. Nuclei were counterstained with DAPI (blue). Bar = 20 μm. (C, D) Representative immunofluorescence staining images of p‐RIP3, p‐MLKL (Red) and GFAP (green) in cortex and hippocampal DG, CA1 and CA3 regions. Nuclei were counterstained with DAPI (blue). Bar = 20 μm. (E, F) Representative immunofluorescence staining images of p‐RIP3, p‐MLKL (red) and Iba‐1 (green) in cortex and hippocampal DG, CA1 and CA3 regions. Nuclei were counterstained with DAPI (blue). Right panel: enlargements of images in the boxes in the left panel. Bar = 20 μm

To investigate the cell types with activated p‐RIP3 and p‐MLKL after hydrocephalus, we used double immunofluorescence staining to observe the cellular localization of p‐RIP3 and p‐MLKL in three kinds of cells, including NeuN^+^ neurons, Iba‐1^+^ microglia and GFAP^+^ astrocytes, in the cortex and hippocampal DG, CA1 and CA3 regions. We found that p‐RIP3 and p‐MLKL were mainly colocalized with NeuN+neurons in the hippocampus and cortex after hydrocephalus induction (Figure 3A,B). We also found that p‐RIP3 and p‐MLKL rarely colocalized with Iba‐1^+^ microglia or GFAP^+^ astrocytes at the same time point (Figure 3C‐F).

### 3.4 Nec‐1 and GSK872 reduce necrotic cell death and attenuate the levels of phosphorylated RIP3 and MLKL in the cortex and hippocampus after hydrocephalus

**FIGURE 4:**
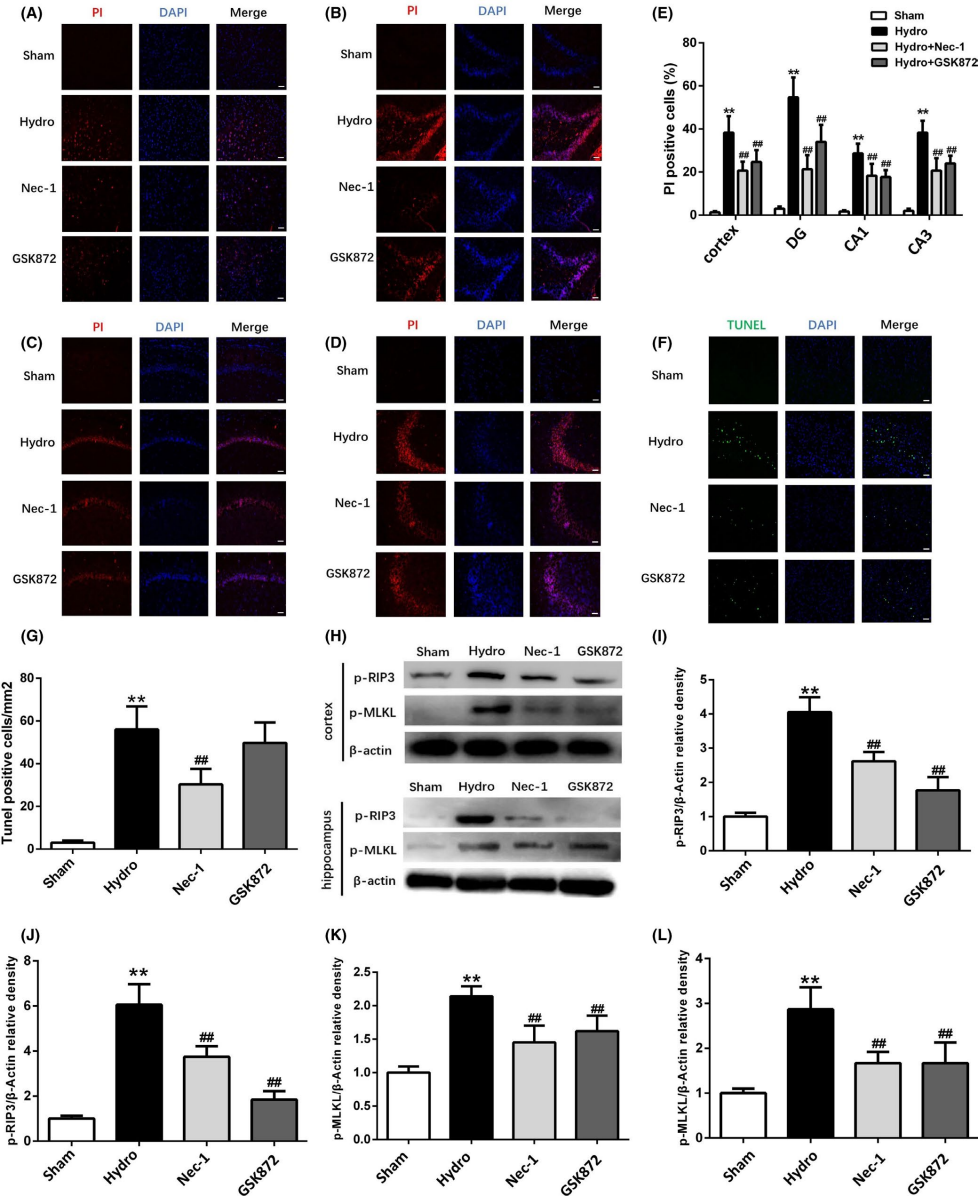
The effect of Nec‐1 and GSK872 on necrotic cell death, the number of TUNEL‐positive cells and the expression of p‐RIP3 and p‐MLKL after hydrocephalus. (A‐D) Representative fluorescence micrographs of PI labelling (red) in sham group, hydrocephalus group, Nec‐1 group and GSK872 group. (E) The number of PI‐positive cells changes in each group. Nuclei were counterstained with DAPI (blue). Bar = 50 μm. (F) Representative micrographs of TUNEL staining in cortex in each group, and (G) Nec‐1 decreased TUNEL‐positive cells in cortex compared with hydrocephalus group. Nuclei were counterstained with DAPI (blue). Bar = 50 μm. (H‐L) Representative Western blotting bands and quantitative analyses of p‐RIP3 and p‐MLKL protein levels in the cortex and hippocampus in each group. n = 6 animals per group. Data are expressed as the mean ± SEM; statistical differences among groups were analysed using Student's *t*‐test; **P < .01 vs. sham. **^##^** *P* < .01 vs. hydrocephalus

PI was impermeable to the intact cell membrane and was used to label necrotic cell death. We investigated whether necroptosis inhibitors Nec‐1 and GSK872 alleviated the number of PI‐positive cells after hydrocephalus. As shown in Figure 4, both Nec‐1 and GSK872 decreased the numbers of PI‐positive cells in the cortex and hippocampus compared with the hydrocephalus group (Figure 4A‐E). Moreover, the number of TUNEL‐positive cells was decreased in the Nec‐1 treatment group compared with the hydrocephalus group (Figure 4F,G), but GSK872 did not have a similar effect. Additionally, Western blotting showed that p‐RIP3 and p‐MLKL expression levels in the cortex and hippocampus were decreased by Nec‐1 and GSK872 compared with the hydrocephalus group (Figure 4H‐L).

### 3.5 Nec‐1 and GSK872 inhibit the activation of microglia and astrocytes and inflammation in the cortex and hippocampus after hydrocephalus

**FIGURE 5:**
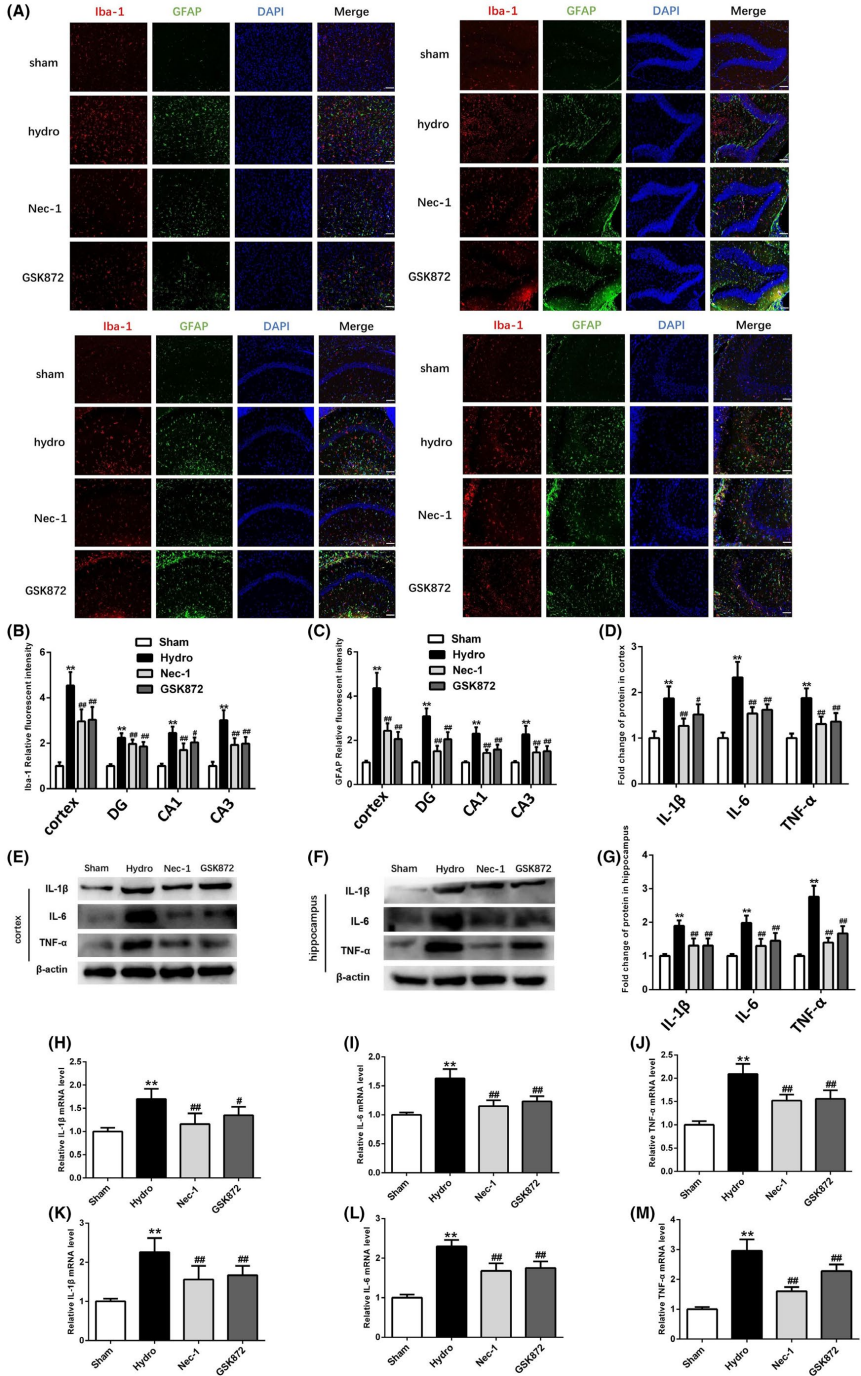
The effects of Nec‐1 and GSK872 on activation of microglia and astrocytes and inflammation after hydrocephalus. (A) Representative immunofluorescence staining images of Iba‐1(red) and GFAP (green) in cortex and hippocampal DG, CA1 and CA3 regions. Nuclei were counterstained with DAPI (blue). Bar = 100 μm. (B, C) Quantitative analyses of Iba‐1 and GFAP relative fluorescent intensity in cortex and hippocampal DG, CA1 and CA3 regions in each group. (D‐G) Representative Western blotting bands and quantitative analyses of IL‐1β, IL‐6 and TNF‐α protein levels in the cortex and hippocampus in each group. (H‐M) Quantitative real‐time PCR analyses of IL‐1β, IL‐6 and TNF‐α mRNA expression in each group. n = 6 animals per group. Data are expressed as the mean ± SEM; statistical differences among groups were analysed using Student's *t*‐test; **P < .01 vs. sham. **^#^** *P* < .05 vs. hydrocephalus. **^##^** *P* < .01 vs. hydrocephalus

On day 28 after kaolin injection, the numbers of GFAP‐positive cells and Iba‐1‐positive cells were increased in both cortex and hippocampus in the hydrocephalus group compared with the sham group. However, fewer GFAP‐positive cells and Iba‐1‐positive cells were noted in the Nec‐1 and GSK872 groups (Figure 5A‐C). Moreover, Western blotting showed that IL‐1β, IL‐6 and TNF‐α expression levels were increased in the hydrocephalus group compared with the sham group, and all of these molecules were suppressed by treatment with Nec‐1 and GSK872 (Figure 5D‐G). Furthermore, the results of RT‐PCR were similar to those of Western blotting (Figure 5H‐M).

### 3.6 Nec‐1 and GSK872 improves neurobehavioral performance slightly and does not significantly reduce the size of the lateral ventricle

**FIGURE 6:**
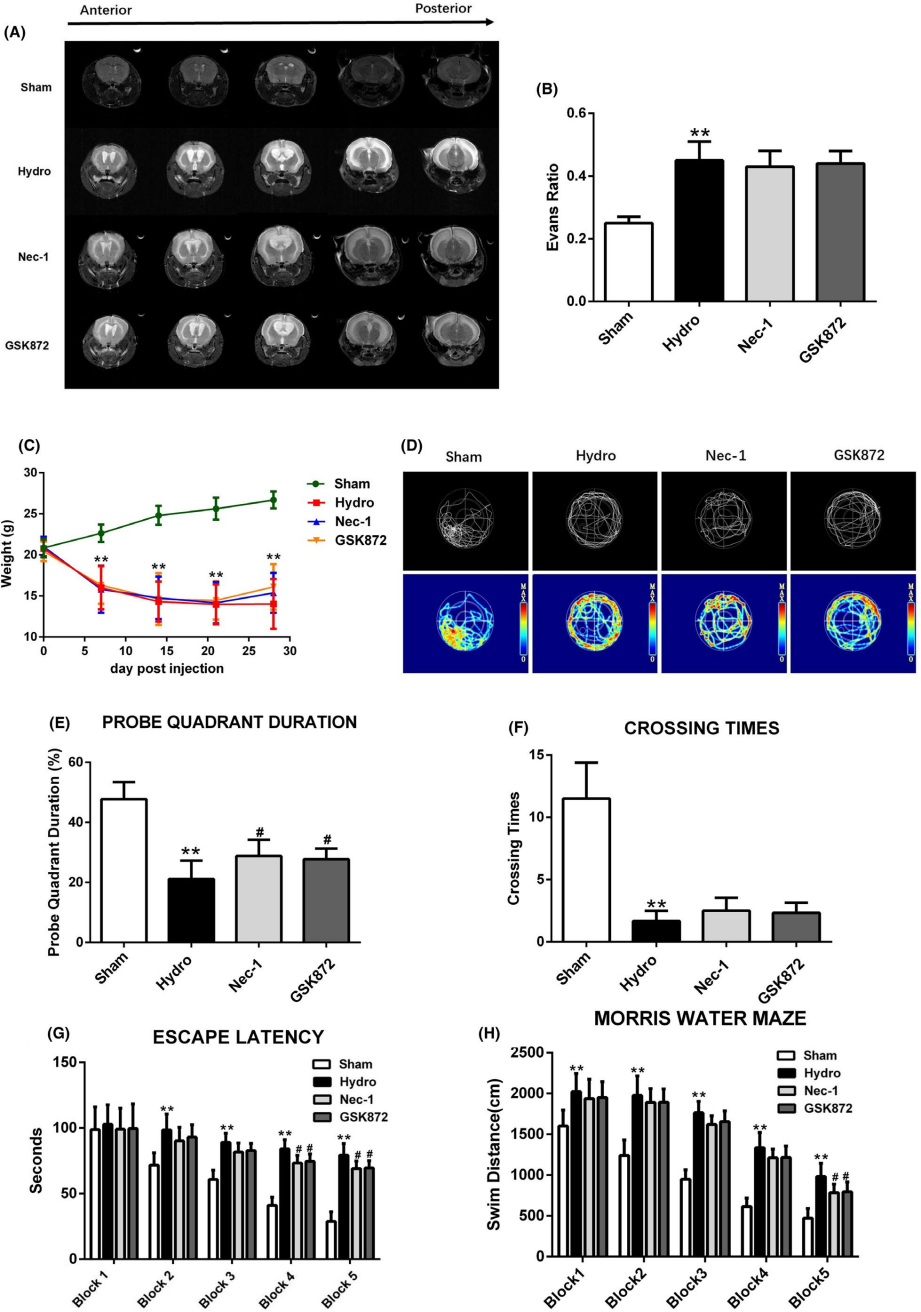
The effects of Nec‐1 and GSK872 on long‐term neurobehavioural outcomes after hydrocephalus. (A, B) Representative image of 7.0T MRI and Evans ratio in each group. (C) Changes of body weight in each group at 7, 14, 21, 28 days. (D) Typical traces of Morris water maze on day 28 after hydrocephalus. (E) Probe quadrant duration of Morris water maze on day 28 after hydrocephalus. (F) Crossing times on day 28 after hydrocephalus. (G, H) Escape latency and swim distance of Morris water maze on days 23 to 28 after hydrocephalus. n = 6 animals per group. Data are expressed as the mean ± SEM; Statistical differences among groups were analysed using Student's *t*‐test; **P < .01 vs. sham. **^#^** *P* < .05 vs. hydrocephalus

**FIGURE 7:**
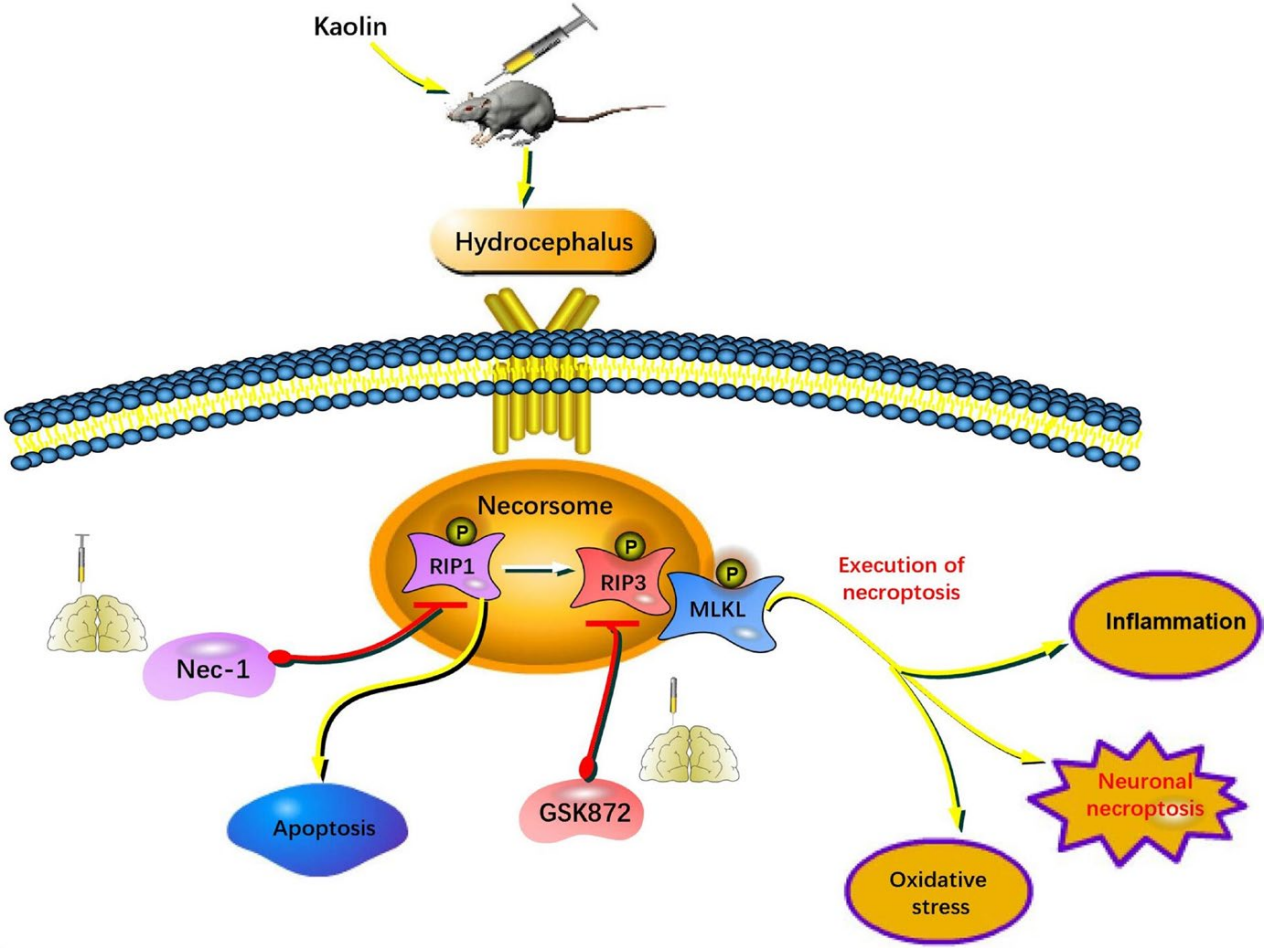
The mechanism of necroptosis after hydrocephalus. After hydrocephalus, RIP1 recruits and promotes RIP3 phosphorylation. Then, a body consisting of RIP1, RIP3 and MLKL is formed. MLKL is phosphorylated by p‐RIP3, located at position 357 of threonine and position 358 of serine, and is considered to be the executor of necroptosis. p‐MLKL finally leads to the exposure of cells by changing permeabilization in plasm membrane and result in necrosis of the cell. However, the effects were improved after the use necroptosis inhibitors Nec‐1 and GSK872

The ventricular volumes were significantly enlarged in the hydrocephalus group compared with the sham group. However, compared with that in the hydrocephalus group, Nec‐1 and GSK872 did not significantly reduce the ventricle volume (Figure 6A,B). Moreover, we found that Nec‐1 and GSK872 could not reverse weight loss in mice after model induction (Figure 6C). Furthermore, we assessed the neurocognitive functions by the MWM test (Figure 6D). In the probe quadrant trial, the mice in the hydrocephalus group spent less time in the target quadrant than those in the sham group, while the reference memory deficits were increased by Nec‐1 and GSK872 treatment (Figure 6E). Moreover, compared with the sham group, the hydrocephalus group performed fewer platform crossings, while the mice in the Nec‐1 and GSK872 groups did not perform significantly more platform crossings (Figure 6F). The escape latencies of mice in the hydrocephalus group were increased compared with those in the sham group. The escape latencies of mice in the Nec‐1 and GSK872 groups were decreased compared with the hydrocephalus group (Figure 6G). The swim distance of mice in the hydrocephalus group was also increased compared with the sham group, and the swim distances of mice in the Nec‐1 and GSK872 groups were decreased compared with the hydrocephalus group (Figure 6H). Working model of necroptosis after hydrocephalus is shown in Figure 7.

## 4 DISCUSSION

Hydrocephalus induced by kaolin is still the most commonly used model to mimic obstructive hydrocephalus.^24^ The present study revealed three major findings: (a) 28 days after modelling, obvious pathological changes and necrotic cell death occurred in the hippocampus and cortex, and the ependymal cilia of mice were seriously damaged; (b) hydrocephalus increased the expression levels of the necroptosis markers p‐RIP3 and p‐MLKL and inflammatory factors in the hippocampus and cortex, and p‐RIP3 and p‐MLKL were primarily located in neurons; (c) treatment with the necroptosis inhibitors Nec‐1 and GSK872 reduced the expression of the necroptosis markers p‐RIP3, p‐MLKL, inflammatory factors and reduced astrocyte and microglial activation. However, the neurobehavioral defects improved slightly and the size of the lateral ventricle did not decrease significantly in response to Nec‐1 and GSK872.

Hydrocephalus compacts the cerebral cortex and hippocampus and changes their output neurons, which is associated with spatial learning and memory impairment.^25^ 28 days is a common time point for observation after kaolin‐induced hydrocephalus.^26^ In this study, we observed obvious pathological damage in the hippocampus and cortex by HE, Nissl and FJB staining 28 days after hydrocephalus modelling, but few TUNEL‐positive cells were observed in the hippocampus. In addition, disruption of ependymal cell cilia results in CSF flow occlusion or failure to regulate the transfer of fluid, ions and small molecules between the cerebral parenchyma and ventricular fluid.^27^ SEM showed that the ependymal cilia were seriously damaged, sparsely arranged and collapsed in the hydrocephalus model at 28 days. These observations indicated significant damage to brain tissue 28 days after the hydrocephalus model was established. Therefore, this time point is the focus of this study.

The detailed mechanisms of cell death or neuronal loss after hydrocephalus remain unclear. Necroptosis has been described as the basis of ischaemic injury, neurodegenerative diseases, viral infection and other diseases.^28^, ^29^, ^30^ The phosphorylation of RIP3 and MLKL plays an important role in the development of necroptosis.^31^ We further found that p‐RIP3 and p‐MLKL expression in the cortex and hippocampus increased after hydrocephalus. Moreover, p‐RIP3 and p‐MLKL were mainly colocalized with neurons but not astrocytes or microglia in the hippocampal cortex after hydrocephalus. Another feature of necroptosis is cell temperature, which can be detected by PI staining. We found that PI‐positive cells were increased in the hippocampus and cortex after hydrocephalus. These results suggest that necroptosis may be involved in cell death after hydrocephalus. This study is the first to report necroptosis in kaolin‐induced hydrocephalus. Emerging evidence suggests that necroptosis can also trigger an inflammatory response.^32^ Targeted interventions in necroptosis may ameliorate inflammatory and neurodegenerative diseases.^33^ Inflammation plays an important role in the occurrence and development of hydrocephalus.^34^, ^35^, ^36^, ^37^ A previous study demonstrated that the expression of the proinflammatory cytokine IL‐1β is significantly increased in the cortex and hippocampus in chronic hydrocephalus in juvenile rats.^38^ In this study, we found that the expression of inflammatory cytokines such as IL‐1β, IL‐6 and TNF‐α was increased in the hippocampus and cortex in mice in the hydrocephalus group, indicating that inflammation is closely associated with the occurrence and development of kaolin‐induced hydrocephalus in mice. Further study is required to determine whether inhibiting necroptosis can reduce the inflammatory response after hydrocephalus.

The RIP1 inhibitor Nec‐1 is a specific necroptosis inhibitor and effectively inhibits RIP1‐induced RIP3 activation, which has been shown to rescue cell death and provide neuroprotection in neurodegenerative diseases, include ischaemic stroke, TBI, spinal cord injury (SCI), subarachnoid haemorrhage (SAH) and intracerebral haemorrhage.^39^, ^40^, ^41^, ^42^ In addition, several lines of evidence indicate that RIP3 is a key signalling molecule involved in the necroptosis pathway with multiple roles in cell death and inflammation. For example, increased RIP3 expression contributes to the induction of necroptotic cell death in hippocampal neurons following cerebral ischaemia‐induced inflammatory responses.^43^ A recent study showed that RIP3 ablation provides neuroprotection against dopaminergic neurodegeneration in experimental Parkinson's disease.^44^ GSK872 is a highly specific RIP3 inhibitor that is widely used in the study of necroptosis.^45^ Studies have shown that GSK872 reduces the phosphorylation of RIP3 and MLKL, thereby ameliorating brain injury, antioxidant capacity and proinflammatory cytokines.^46^, ^47^, ^48^, ^49^ RIP3‐ and MLKL‐dependent necrosis exacerbates tissue damage and inflammation in some animal models. We therefore anticipated that necroptosis would play a proinflammatory role after hydrocephalus. In a hydrocephalus mouse model, Nec‐1 and GSK872 reduce necrotic cell death, effectively inhibit the phosphorylation of RIP3 and MLKL, and reduce the levels of inflammatory factors such as IL‐1β, IL‐6 and TNF‐α in the cortex and hippocampus. Moreover, the results showed that Nec‐1 can significantly reduce the number of TUNEL‐positive cells in the cortex after hydrocephalus, whereas GSK872 has no similar effect. Astrogliosis and microgliosis have been reported in kaolin‐induced hydrocephalus in mice, rats and ferrets of different ages.^50^, ^51^ Reactive astrogliosis in the brain is characterized by GFAP upregulation and astrocytic process hypertrophy.^52^ The level of GFAP is elevated in the CSF of human patients, and cortical biopsies of patients with chronic hydrocephalus show increased astrogliosis.^53^, ^54^ Activated microglia are associated with some nervous system diseases, such as Alzheimer's disease and chronic pain.^55^ Previous reports have shown that inhibiting necroptosis can reduce not only the inflammatory response but also the expression of the astrocytic marker GFAP and the microglial marker Iba‐1.^22^, ^56^ Moreover, there is evidence that necroptosis causes neuronal damage by affecting microglia or astrocytes.^57^, ^58^ In this study, we found that microglia and astrocytes were activated in the hippocampus and cortex of mice after hydrocephalus, and this response was alleviated by Nec‐1 and GSK872. We hypothesize that this effect may be associated with the inhibition of the necroptosis‐mediated inflammatory response after hydrocephalus. In addition, we found that both Nec‐1 and GSK872 improved the neurobehaviours of mice slightly after hydrocephalus but had no significant effects on body weight or lateral ventricular enlargement. Taken together, these data indicate that Nec‐1 and GSK872 have certain neuroprotective effects after hydrocephalus, such as anti‐inflammatory effects, but some of the effects of blocking necroptosis on behaviour appear to be relatively minor, suggesting that necroptosis is perhaps not a major contributor to the impact of hydrocephalus on behaviour.

There are some limitations in the present study. First, we focused on one time point and did not observe potential changes at different time intervals after hydrocephalus. Second, we only showed pharmacological inhibition of necroptosis, and genetic inhibition was not further verified in gene knockout mice, such as RIP3^‐/‐^ and MLKL^‐/‐^ mice. More accurate results may be obtained by gene inhibition. Additionally, one of the major limitations is the descriptive nature of the experiments, which did not explore the mechanism by which hydrocephalus causes necroptosis. Therefore, these issues need to be clarified in future studies.

In summary, in line with increasing evidence of neuroprotection after inhibition of necroptosis, we found that necroptosis occurs after hydrocephalus. Although necroptosis may not be the major mode of cell death after hydrocephalus, inhibition of necroptosis may still produce some neuroprotective effects. Therefore, reducing necroptosis has potential value for the management of hydrocephalus.

## ETHICAL APPROVAL AND CONSENT TO PARTICIPATE

All experiments were reviewed and approved by the Ethics Committee of Sichuan University.

## CONFLICT OF INTEREST

The authors declare no conflict of interest. The sponsors had no role in the design, execution, interpretation or writing of the study, or in the decision to publish the results.

## AUTHOR CONTRIBUTIONS

Conceptualization, Chang Liu and Yaxing Chen; Methodology and Software, Wenyao Cui, Yi Cao, Long Zhao, Shuangmin Fan; Validation, Xiaoyin Liu, Keru Huang; Data Analysis, Haoxiang Wang; Data; Writing – Project Administration, Liangxue Zhou, Aiping Tong.

## Data Availability

The data that support the findings of this study are available from the corresponding author upon reasonable request.
